# An evaluation of the Coulter DACOS analyser

**DOI:** 10.1155/S1463924686000147

**Published:** 1986

**Authors:** A. E. Hurrell, R. A. Hall, J. L. Robinson

**Affiliations:** Department of Biochemistry Good Hope Hospital West Midlands Sutton Coldfield B75 7RR UK


Journal of Automatic Chemistry/*Journal of Clinical Laboratory Automation*, Vol. 8, No. 2 (April–June 1986), pp. 63–69

### An evaluation of the Coulter DACOS analyser

**A. E. Hurrell, R. A. Hall and J. L. Robinson**

*Department of Biochemistry, Good Hope Hospital, Sutton Coldfield, West Midlands B75 7RR, UK*

### Introduction

The Coulter DACOS (Discrete Analyser with Continuous Optical Scanning) is a discrete analyser capable of producing 450 test results/h. It has a repertoire of 28 chemistries, which use small volumes of both serum (2 μl to 20 μl) and reagents (20 μl to 300 μl). The minimum volume which can be read by the spectrophotometer is 120 μl.

Two independent groups of workers in the UK developed novel prototype analytical systems [1 and 2]. They sought to incorporate the advantages of centrifugal analysis [3], such as frequent optical measurements, with the benefits of sequential continuous analysis. The main feature of the resulting system is that instead of samples being spun in a rotor around the light source, the optical system itself is rotated continuously around the samples.

As a result, the instrument has been developed into a high-capacity discretionary analyser. It has a greater throughput and less operator involvement than the centrifugal analyser, which has generally been used to handle the work-load in separate batches of tests.

The DACOS incorporates a minicomputer which is used for instrument control, reaction monitoring and data processing. Commercial development of the system has been carried out by Coulter Electronics Ltd (Northwell Drive, Luton, Bedfordshire LU3 3RH, UK) and Coulter Electronics, Inc. (Hialeh, Florida, USA).

### Instrument operation

### *Description*

The DACOS is a modular system consisting of the following units: power supply, system control unit with a cartridge disk system, analyser, video display terminal with keyboard and printer.

Test programming is carried out via the keyboard. A sequence number and details of the tests to be performed are entered for each specimen. Complete demographic data may be entered if required. This information is filed by the central processing unit (CPU) and is linked to the sample position on a particular tray. The tray, which has a maximum of 64 sample positions, is loaded into the DACOS and the analyses are performed. All the tests requested for any one sample are performed before moving to the next specimen.

The instrument contains a number of dedicated microprocessors. These control pipetting, via Hamilton (Hamilton Bonadurz AG, PO Box 26, CH 7402 Bonadurz, Switzerland) syringes linked to stepping motors, sample and reagent arm movement, cuvette and sample disk turntable indexing and operation of the cuvette wash system.

Instrument functions occur on an 8 s time cycle. The processes of sample addition, reagent 1 and 2 addition, spectrophotometry and cuvette washing operate sequentially on each sample within the instrument. Maximum sample incubation time is 12·5 min for a single reagent test or 9·5 min if a second reagent is to be added.

The plastic cuvettes, which are contained within a 120-place rotor, can be reused and individually replaced. They have been found to have a life-span of three to four months in routine use. However, staining by some reagents can considerably reduce their life-span.

In operation, serum is sampled from a disposable sample plate. This has 64 patient and 16 'stat' wells, each of which have a maximum volume of 350 μl, together with 16 calibrator/control wells, each of which have a maximum volume of 700 μl. The serum is washed into the cuvette ring with 20 to 200 μl of saline. Mixing is accomplished by force of addition of the sample and diluent.

Provided sufficient volume is present (120 μl), a blank reading may be made on the solution before addition of the first reagent after 42 s. Mixing is accomplished afterwards by vibration of the reagent probe within the cuvette. A blank reading for the combined sample versus reagent may then be taken. If a second reagent is required, it can be added and mixed after a further 160 s.

Readings can then be taken every 8 s for up to 12·5 min with a one-reagent chemistry (92 data points), or for 9·5 min with a two reagent chemistry (71 data points).

After the reaction has been completed, the cuvette is laundered and its optical cleanliness checked ready for the next serum addition.

The complete operation on the instrument is computer controlled by a Texas Instruments (Texas Instruments Corporation, PO Box 2909, Austin, Texas 78769, USA) computer, Model 990. Data storage (10 megabytes) is on cartridge type disks. Five megabytes of this are in the form of a removable disk and the system is provided with a number of disk options; amongst which is a test definition disk which allows the operator to program the various test parameters required by the system.

*Calibration*

The DACOS can be calibrated by one of several different methods. Each analyte can use as many as six standards of different concentration and each standard point can be determined by a maximum of three replicates.

The simplest calibration method is for enzyme analysis. This incorporates a blank, where water is substituted for sample, to check the performance of the reagents. Results are calculated by means of the appropriate molar extinction coefficient, which has previously been entered into memory.

Most of the chemistries are calibrated by using a water blank and three serum standards. A measure of quality control is obtained by applying linear regression analysis to the standard line. This monitors linearity, slope and intercept. The chi-square test is used to indicate the variability of the standard points about the computed line.

More complex analyses, such as EMIT (EMIT Trade Mark of Syva Company, PO Box 10058, Palo Alto, California 94303, USA), make use of the maximum six standards. Their absorbances are plotted by means of a five-parameter LOGIT curve fitting process.

In practice, the test instrument has been calibrated at the beginning of each working day by means of the DACAL calibration system marketed by Coulter. This comprises three calibrators of 3 ml each. For the sake of economy, each calibrator is divided into three aliquots of 1 ml after reconstitution and the aliquots are frozen, one being thawed for use each day. Comparison of results has shown that this procedure is satisfactory.

*Result calculations*

After the operator is satisfied that the instrument is correctly calibrated, test samples may be run and the absorbance readings passed through a series of data processing algorithms.

The tests programmed into the DACOS fit one of the following four categories:

(1) Linear rate of absorbance change with time (zero order kinetics).
(2) Exponential change of absorbance over a fixed time period (first order kinetics).
(3) Change in absorbance over a fixed interval of time (initial reaction rate).
(4) Final absorbance when reaction is complete (equilibrium reaction).

The output from the DACOS is from a Data General (FACIT, 235 Main Dunstable Road, Nashua, New Hampshire 03061, USA) printer onto standard listing paper. The software includes quality-control procedures and the ability to generate duplicate reports if required. An RS232-C interface is standard equipment on the instrument and a bidirectional interface will shortly be available.

### The evaluation

An initial evaluation was carried out over seven days. The instrument performed close to specification. However, two particular features were identified as requiring further investigation: excessive noise and sample-to-sample interaction.

Subsequently, an evaluation of a variety of methods was carried out over a three-month period. The guidelines adopted for the evaluation were taken from those published by Broughton *et al.* [4] and the European Committee for Clinical Laboratory Standards [5].

### Materials and methods

*Instruments and reagents*

The DACOS analyser was purchased from COULTER Electronics Ltd, as were DART(R) reagents for glucose and uric acid assays, together with DACAL lyophilized human serum calibrators. For glucose and uric acid tests the reagents and calibrators were used as directed by the manufacturer. The remainder of the methods – ALT, ALP, GGT, CK, LDH (Non-standard abbreviations: ALT, alanine-amino transferase [EC 2.6.1.1]; ALP, alkaline phosphatase [EC 3.1.3.1]; GGT, gamma glutamyltransferase [EC 2.3.2.2]; CK, creatine kinase [EC 2.7.3.2]; LDH, lactate dehydrogenase [EC 1.1.1.27]) – used during the evaluation, with the exception of

*Table 1. Methods used.*

| Analyte | Sample volume (μl) | Diluent volume (μl) | Reagent Volumes (μl) 1 | 2 | Supplier | Method | Catalogue No. |
|---|---|---|---|---|---|---|---|
| Cholesterol | 2 | 120 | 200 | — | BCL | CHOD/PAP | 236691 |
| Glucose (BCL) | 3 | 120 | 300 | — | BCL | GOD/PAP | 166391 |
| Glucose (DART) | 4 | 120 | 200 | — | DART | Hexokinase | — |
| Triglyceride | 2 | 120 | 200 | — | MERK | GK/NAD/INT | — |
| Uric acid (BCL) | 4 | 120 | 150 | — | BCL | Uricase/PAP | 620994 |
| Uric acid (DART) | 6 | 120 | — | 80 | DART | Uricase/PAP | — |
| ALAT | 20 | 80 | 150 | 20 | BCL | SCE | 191345 |
| ALP | 3 | 20 | 150 | 20 | BCL | DGFKC | 415286/415294 |
| CK | 6 | 40 | 100 | 20 | BCL | DGFKC/SCE | 475742/476769 |
| GGT | 8 | 40 | 100 | 20 | BCL | 3-Carb-4-Nitro | 543098/543101 |
| LDH | 4 | 20 | 100 | 20 | BCL | DGFKC | 543047/543063 |

triglyceride, were based on BCL (Boehringer Corporation Ltd, Bell Lane, Lewes, East Sussex, UK) kits as specified in table 1. Triglyceride was measured using Merck reagents supplied by E. M. Diagnostics (E. M. Diagnostics, Broom Road, Poole, Dorset, UK). In all cases these reagents were under-diluted to accommodate the addition of saline diluent as required by the DACOS sampling system.

The instruments used for comparison studies were LKB 8600 reaction rate analysers (LKB Instruments Ltd, South Croydon, Surrey, UK), for the enzymes, Technicon AAII analysers for cholesterol, triglyceride and uric acid (Technicon Instruments Ltd, Basingstoke, Hampshire, UK); and a Beckman Glucose 2 (Beckman-RIIC Ltd, High Wycombe, Buckinghamshire, UK) analyser for glucose. The reagents used on the comparative instruments were of the same origin and composition as those on the DACOS. The two exceptions to this were glucose reagent from Beckman and uric acid reagent from BCL (cat. No. 157104).

### *Specimens*

Over 200 serum specimens which had been submitted for routine analysis were analysed on the DACOS and the appropriate comparative instrument for the analytes requested. The sera were selected to give a spread of concentrations covering low, medium and high values.

### *Control sera*

M + D QAP Chemistry Controls Levels I and II, Merz Dade (American Hospital Supply (UK) Ltd, Didcot, Oxfordshire, UK), BCL Precinorm U, Precipath U, Precilip EL, and Beckman Decision were used in the study.

### *Calibration*

The Technicon AAII chemistries were calibrated using Technicon serum calibrators. The Beckman Glucose 2 analyser was calibrated using the Beckman 150/50 standard supplied with the company's glucose reagents.

### Results

### *Accuracy*

The results from the DACOS were compared with existing methods by the Deeming method of correlation [6]. Results are shown in table 2. Quality-control material was made available from the National Quality Control Scheme (Wolfson Research Laboratories, Q.E. Medical Centre, Birmingham, UK), and table 3 shows a comparison of the results obtained from this and from the Wellcome (Wellcome Diagnostics, Dartford, Kent, UK) clinical chemistry control scheme.

*Table 2. Correlation with existing methods.*

| Analyte | N | Correlation coefficient | Slope | Intercept |
|---|---|---|---|---|
| Cholesterol | 100 | 0·969 | 1·098 | −0·02 |
| Glucose (BCL) | 200 | 0·997 | 1·003 | −0·09 |
| Glucose (DART) | 200 | 0·996 | 0·969 | 0·07 |
| Triglyceride | 100 | 0·989 | 1·065 | −0·04 |
| Uric acid (BCL) | 200 | 0·953 | 0·917 | −42·50 |
| Uric acid (DART) | 200 | 0·970 | 0·952 | −32·39 |
| ALAT | 200 | 0·997 | 1·029 | −4·68 |
| ALP | 200 | 0·998 | 1·076 | 0·07 |
| CK | 199 | 0·995 | 1·102 | −18·31 |
| GGT | 200 | 0·997 | 1·000 | 0·85 |
| LDH | 199 | 0·978 | 1·129 | 35·91 |

*Table 3. Assessment of accuracy. Comparison with known control sera from Wellcome and NQAS schemes.*

| Analyte | No. analysed | Concentration | Mean difference from true value | |
|---|---|---|---|---|
| | | Range | Units | % |
| Cholesterol | 15 | 2·15–5·70 | +0·012 | +4·59 |
| Glucose (BCL) | 15 | 3·91–12·93 | +0·034 | +0·45 |
| Glucose (DART) | 15 | 3·91–13·04 | −0·040 | −0·17 |
| Triglyceride | 6 | 0·51–1·60 | +0·098 | +11·4 |
| Uric acid (BCL) | 15 | 225·0–598·0 | −39·9 | −10·3 |
| Uric acid (DART) | 15 | 225·0–598·0 | −24·1 | −7·58 |
| ALAT | 5 | 26·5–121·7 | −4·88 | −8·30 |
| ALP | 5 | 111·0–470·0 | +31·6 | +12·6 |
| CK | 4 | 57·1–530·5 | −30·3 | −8·74 |
| GGT | 4 | 25·1–98·0 | −2·113 | −5·69 |
| LDH | Insufficient data available | | | |

*Table 4. Within-batch precision.*

| | | Level 1 | | Level 2 | | Level 3 | |
|---|---|---|---|---|---|---|---|
| Analyte | Units | Mean | CV | Mean | CV | Mean | CV |
| Cholesterol | mmol/l | 2·47 | 1·1 | 6·55 | 1·6 | 9·90 | 1·5 |
| Glucose (BCL) | mmol/l | 2·47 | 0·9 | 6·16 | 1·2 | 13·95 | 1·1 |
| Glucose (Dart) | mmol/l | 2·40 | 0·8 | 6·09 | 0·9 | 13·86 | 0·8 |
| Triglyceride | mmol/l | 0·77 | 1·5 | 1·72 | 1·1 | 3·27 | 1·3 |
| Uric acid (BCL) | µmol/L | 144·03 | 1·2 | 224·06 | 1·2 | 471·08 | 1·2 |
| Uric acid (Dart) | µmol/L | 162·07 | 1·1 | 224·02 | 0·9 | 500·09 | 1·2 |
| ALAT | IU/l | 29·09 | 2·0 | 78·07 | 2·0 | 104·04 | 1·6 |
| ALP | IU/l | 92·01 | 3·6 | 463·07 | 1·2 | 876·07 | 1·1 |
| GGT | IU/l | 34·09 | 3·1 | 90·05 | 2·5 | 210·01 | 1·8 |
| CK | IU/l | 54·06 | 2·3 | 187·09 | 1·2 | 441·03 | 0·6 |
| LDH | IU/l | 101·01 | 3·9 | 177·01 | 2·2 | 327·05 | 2·3 |

*Table 5. Between-batch precision.*

| | | Level 1 | | Level 2 | | Level 3 | |
|---|---|---|---|---|---|---|---|
| Analyte | Units | Mean | CV | Mean | CV | Mean | CV |
| Cholesterol | mmol/l | 2·63 | 2·9 | 6·41 | 2·1 | 10·67 | 2·1 |
| Glucose (BCL) | mmol/l | 4·63 | 2·3 | 6·21 | 1·9 | 13·92 | 1·7 |
| Glucose (Dart) | mmol/l | 4·54 | 2·9 | 6·59 | 2·7 | 13·90 | 2·6 |
| Tryglyceride | mmol/l | 1·01 | 4·0 | 1·70 | 4·7 | 3·38 | 5·0 |
| Uric acid (BCL) | µmol/l | 255·3 | 4·3 | 433·4 | 3·5 | 472·01 | 3·3 |
| Uric acid (Dart) | µmol/l | 270·3 | 3·2 | 456·9 | 3·2 | 500·03 | 3·1 |
| ALAT | IU/l | 31·3 | 3·9 | 52·8 | 2·1 | 100·01 | 2·1 |
| ALP | IU/l | 150·2 | 3·9 | 454·6 | 4·3 | 865·05 | 1·9 |
| GGT | IU/l | 34·4 | 4·3 | 66·9 | 2·5 | 211·08 | 1·8 |
| CK | IU/l | 53·1 | 9·5 | 145·9 | 2·6 | 385·04 | 6·5 |
| LDH | IU/l | 142·9 | 7·0 | 316·2 | 3·6 | 346·05 | 3·3 |

*Table 6. Linearity ranges.*

| Analyte | Unit | Programmed linearity | Laboratory reference interval |
|---|---|---|---|
| Cholesterol | mmol/l | 12·0 | less than 7·3 |
| Glucose (BCL) | mmol/l | 30·0 | 3·0–5·0 |
| Glucose (Dart) | mmol/l | 33·0 | 3·7–5·9 |
| Tryglyceride | mmol/l | 10·0 | less than 1·8 |
| Uric acid (BCL) | µmol/l | 1200·0 | 110·0–360·0 |
| Uric acid (Dart) | µmol/l | 1490·0 | 140·0–490·0 |
| ALAT | IU/l | 1000·0 | less than 30 |
| ALP | IU/l | 2500·0 | less than 275 |
| GGT | IU/l | 1000·0 | less than 50 |
| CK | IU/l | 2500·0 | less than 170 |
| LDH | IU/l | 1000·0 | less than 22 |

### *Precision*

The within-batch precision was determined by analysing 20 identical serum samples for the same test consecutively, at three different levels. The results obtained are summarised in table 4.

The between-batch precision was determined from three different levels of control sera which were analysed on 20 consecutive working days. The results obtained are summarized in table 5.

### *Linearity*

The ranges obtained from linearity studies are shown in table 6. Only a very small number of tests were lost as a result of being over-range; fewer than 1% needed to be repeated using a diluted serum. The main tests affected in this way were the enzymes. Since the chemistries may be defined entirely by the user, taking as many or as few data points as he requires for each analysis, linearity ranges can effectively be extended to infinity. However, if very high linearity limits were defined, this would obviously result in some loss of sensitivity at low concentrations.

### *Carry-over*

*Sample-to-sample:* sample cross-contamination [7] was determined by alternately sampling from Eosin (5% in water) and distilled water, measuring the resulting absorbance change in the water. No increase in absorbance was found and it was concluded that no sample cross-contamination could be demonstrated.

The Broughton procedure [7] was used to assess carry-over between tests: sampling of three specimens with a high concentration of a particular analyte was followed by sampling of three specimens with a low concentration. The experiment was repeated 20 times for each of the analytes under investigation. The carry-over for all the chemistries was less than 0·5%.

Finally, the effect on carry-over of sampling different volumes was determined [8]. In order to exploit the versatility of the DACOS and use it in a true random access mode, it is possible for a test using a high volume of serum (for example 20 μl) to be followed by a test using a relatively low volume of serum (for example 3 μl).

A typical experiment involved sampling three acid phosphatase estimations (20 μl sample volume), from a serum containing a considerably raised alkaline phosphatase, followed by three estimations for alkaline phosphatase, b1, b2, b3 (sample volume 3 μl) from a serum containing low activity of that analyte. The resulting errors were calculated by using the following formula, which is a modification of that of Broughton [7]:

$$\text{Sampling error} = \frac{b1 - b3}{a - b3} \times 100\%$$

where b1 is the first alkaline phosphatase result and b3 is the last alkaline phosphatase result. a is the mean value, from five estimations, of the concentration of alkaline phosphatase in the sample used for acid phosphatase estimation. This experiment indicated a sampling error of 2·2%. Subsequent modifications by the manufacturer have reduced this error to 0·8%.

*Reagent-to-reagent:* It was thought that in a system where only one reagent probe was used to sample from several reagent containers, the possibility existed of transferring reagent from one container to another.

Reagent cross-contamination was determined in a similar manner to the sample-to-sample cross-contamination, in that 5% Eosin solution was used as one reagent and was followed by distilled water as the other. After measuring the absorbance of the distilled water, the cross-contamination was found to be approximately one part in eight million from reagent-container to reagent-container.

Reagent-to-reagent carry-over in the reaction cuvette was again determined by the use of Eosin solution [8]. After allowance for volume and dilution effects, this carry-over was found to be approximately one part in 8000.

### *Sample evaporation*

Evaporation from the sample plate was assessed in two ways:

(1) By repeatedly weighing the plate with the filled sample cups in position, the loss from evaporation was found to be 0·6% after 1 h, 1·1% after 2 h, 2·0% after 4 h and 5·0% after 8 h.

(2) By repeatedly analysing the samples on the plate for glucose, the mean apparent increase in their concentration was found to be 1·2% after 1 h, 2·5% after 2 h, 3·8% after 4 h and 6·5% after 8 h. It was also noted that the precision of the analyses diminished over this time, the coefficient of variation increasing from 1·05% at time zero to 2·18% after 8 h. This suggests that evaporation had been greater from particular areas of the sample plate.

### *Pipette accuracy*

Pipetting accuracy was measured by weighing pipetted amounts of water at different volume settings. Pipettes were found to deliver approximately 3% less than the stated volume. This is not important for the chemistries where standards and samples are treated alike. It could, however, affect enzyme results, which are calculated by means of a factor which includes pipetted volumes. This error could be corrected by the insertion of an additional factor in the chemistry parameter programming.

### Safety

A suspension of *B. subtilis var globigii* was pipetted from a sample plate and used as a biological marker. Considerable splashing of the top cover of the instrument was found to occur, which would necessitate decontamination of the instrument each day with a bacteriocide.

However, air sampling detected no microbiological contamination, so aerosols do not seem to constitute a hazard to the operator.

With respect to mechanical safety, it would be possible, if extremely unlikely, for an operator to trap a finger beneath the sample, reagent and work-station probes. All electrical circuitry and components have been sensibly sited so that any fluid leaks would not enter into high voltage areas.

### Discussion

The purpose of this evaluation was to determine the analytical performance of the DACOS in a clinical laboratory. It was assessed as a replacement for the analytical systems previously used to measure the analytes selected for evaluation. The work-load averaged 200 patient samples per day, or approximately 500 requested tests.

### *Technical performance*

*Accuracy*: all test methods showed an acceptable correlation between the DACOS and existing techniques. The manufacturer's values for the Dacal calibrators were used during the evaluation. Subsequently, recalibration of these calibrators was shown to be the cause of the proportional errors in cholesterol, triglyceride and uric acid analyses. Since the molecular extinction coefficient was used to calculate enzyme results, it is postulated that small methodological differences or pipetting inaccuracy caused the proportional errors in these tests.

*Precision:* the precision data given in tables 4 and 5 show that the DACOS gave acceptable precision when compared with Tonks criteria [9], for the analytes evaluated using the stated calibration and control procedures. The between-batch precision figures for CK, however, were a source of concern. Minor methodological changes have subsequently reduced the CV to 3·4% at a level of 220 IU/l and 2·3% at a level of 355 IU/l respectively.

*Calibration and start up procedures*

There are a number of 'states' in which the DACOS can be left. These range from 'ready', when analysis will begin immediately on request, to 'off', when approximately 15 min are required for arm/tray alignment etc., before the 'ready' state is reached. The operator is able to control the state in which the instrument is left and consequently the length of time required before analysis can begin. Laboratory policy and test/reagent stability will dictate when calibration is required. A procedure of calibrating the instrument once at the beginning of each working day was adopted. With the tests which were used during the evaluation, this resulted in the first 30 min or so of each working day being set aside for calibration. However, 'Stat' analysis facilities remain available during the calibration period. Calibration absorbance data is recorded on the system each time a calibration is performed. This has shown that daily calibration of the enzyme and some of the chemistry channels, particularly cholesterol, triglycerides and glucose, appears to be unnecessary and could be replaced by weekly calibration. Similarly, it has shown that the uric acid channel needed to be calibrated every day.

*Maintenance*

Maintenance of the DACOS is easily performed. Weekly and monthly maintenance checks are performed by laboratory staff and require 30 min and 1 h respectively. Quarterly maintenance is provided by Coulter and takes approximately half a working day.

*Speed and potential throughput*

At first the authors were disappointed to find that the speed of reporting results was dependent upon the number of processes being carried out on the DACOS's main computer. This meant that if it was carrying out a high work-load, i.e. test programming, sample analysis and result output simultaneously, there was a noticeable slowing of screen response and printed result output. Recently, however, a further 256K of memory has been added to the computer system and all functions can be carried out simultaneously, without any noticeable slowing of the system. The printing of results commences approximately 16 min after the start of processing.

If the DACOS was to be used as a profiling instrument, assuming a 12-test profile, 200 samples could be processed in a working day. This represents a throughput of 2500 tests per day. However, the maximum number of samples that could be run if the test to sample ratio was 2:1 would be approximately 500, because of the time required for sample/test programming and print-out. This would represent about 1000 tests per day.

*Sample and reagent volumes*

The small sample volumes required make the instrument very suitable for analysing paediatric specimens. Analyses typically use 4 µl of sample. It was found experimentally that there was a dead volume of 40 µl in the specimen cup, but this can be adjusted to as little as 25 µl. Reagent volumes are also economical and average 100–150 µl. However, reagents which are supplied by companies

*Sample and reagent volumes*

The small sample volumes required make the instrument very suitable for analysing paediatric specimens. Analyses typically use 4 µl of sample. It was found experimentally that there was a dead volume of 40 µl in the specimen cup, but this can be adjusted to as little as 25 µl. Reagent volumes are also economical and average 100–150 µl. However, reagents which are supplied by companies
other than Coulter had to be diluted rather less than usual, in order to account for the addition of the saline diluent during analysis. A sample blank requires the addition of 120 µl of saline. During the preliminary evaluation it was found that certain of the Coulter DART reagents were packaged inappropriately. This applied particularly to albumin and phosphate packs, where the volume of reagent provided was insufficient to complete the analysis of a full tray of 64 samples.

*Stat analyses*

Facilities for emergency assays are extremely good. Any test provided on the system can be processed as a stat analysis. Stat requests made during a routine run are processed as soon as the current specimen has been sampled for all of its assays. These results are available approximately 12 min after the test has been started.

*Interferences*

The effect of possible interferences was tested by analysing serum with and without added haemoglobin, lipid turbidity and bilirubin. No serious alteration of the results was noted for any of the analytes under evaluation. It is therefore concluded that the ability of the DACOS to perform a sample blank correction on every sample processed avoids interference effects.

*Computing*

The DACOS can be equipped with a standard RS232C interface, but only unidirectional (instrument-to-host) communication is available at present. Coulter will be introducing a bidirectional interface in the near future; this was recommended in the DHSS evaluation carried out by Levin *et al.* [10].

*Staffing/staff training*

The introduction of the DACOS has released one operator for other duties. Staff have found it easy to use and need only two or three days' training to become proficient at operating the system.

*Limitations*

*Carry-over:* both during the preliminary evaluation and subsequently during the main evaluation, sample-to-sample interaction was shown to be a problem with the DACOS. The figure of 2·2% interaction, produced in the differential volume experiment, was considered to be excessive. However, Coulter have now introduced a modification which washes the sample probe with far more saline diluent between samples. Repeating the differential volume experiment has produced a value for sample interaction of 0·8%, which is acceptable for clinical purposes.

*Noise*: emission of noise was considered to be excessive during the preliminary evaluation, but a modification to the cuvette wash system has reduced this to an acceptable level, with no appreciable drop in analytical performance.

*Water*: the system uses approximately 11·2 l of type 2 deionized water/running hour. This could be considered to be a limitation. However, it is far more cost effective to launder semi-disposable cuvettes than to purchase disposable plastic cuvettes.

*Reliability*: several persistent problems during the evaluation were encountered, which, although they were corrected extremely quickly and efficiently by Coulter, nevertheless gave rise to some concern about the instrument's long-term reliability. Most of these problems were of a mechanical nature and could well have been caused by the installation of a DACOS unit which had received no checking, following its shipment from the USA. In response to these problems, Coulter now have a policy of checking each unit for up to a month before delivering it to the customer. There were a number of intractable problems related to limitations of the software. The version II software should be specified for all future purchases of the DACOS instrument.

*Electrolyte capability:* direct reading sodium and potassium ion selective electrodes are now available for the DACOS, but were not available in time for the evaluation.

*Sample and reagent volumes*: there are only six pre-set sample volumes available on the DACOS. Similar restrictions apply to reagent volumes, but this has presented few problems in adapting methodologies for use on the instrument.

### Conclusion

The specification and performance of the DACOS is impressive, although the authors have reservations about the instrument's reliability. Analytical results have been consistently accurate and precise, while operator productivity has increased. The introduction of a continuous discrete analysis system in place of methods of batch analysis has resulted in the laboratory providing a better service to the clinicians. Turn-around times on the analyses involved have been reduced by approximately half. Sample throughput is excellent with the DACOS and would satisfy the requirements of most small- to medium-sized district general hospitals. It could also be useful for removing the more expensive assays, such as enzymes, from the test profile provided by larger laboratories. With the introduction of electrolyte analyses, bidirectional interfacing and software revisions, the instrument could be considered as a replacement for traditional profiling techniques.

### Acknowledgements

We would like to thank Mr P. M. G. Broughton, Miss H. Barrington, Mr R. Lawrence and Mr I. Barr for their help in the preparation of this manuscript, and to Coulter Electronics Ltd for their assistance. Our thanks also go to the organizer of the National Quality Control Scheme for providing samples for analysis.

### Addendum

Since completing the evaluation we have had the opportunity to try a new version of the software (1.3.5). This cured many of the reliability problems that we had encountered. However, we then tried using the electrolyte (I.S.E.) unit and, when this was added to the existing system, a number of new computer-orientated problems arose. This finding supports our earlier conclusion that the computer software for the DACOS needs further development and evaluation.

### References

1. Greaves, S. G. *et al.*, Patent Specification Numbers 1491879 and 1491880 (Filed 1973).
2. Brown, S. S. and Mitchell, F. L., National Bureau of Standards, Special Publication 422 (1976), 837.
3. Price, C. P. and Spencer, K. (Eds), *Centrifugal Analysers in Clinical Chemistry* (Praeger, Eastbourne, 1980), p 3.
4. Broughton, P. M. G. *et al.*, *Annals of Clinical Biochemistry,* **11** (1974), 207.
5. *2nd Draft Guidelines for the Evaluation of Analysers in Clinical Chemistry, European Committee for Clinical Laboratory Standards*, Document Vol. 4, No. 1 (1984).
6. Cornbleet, P. J. and Gochman, N., *Clinical Chemistry,* **25/3** (1979), 432.
7. Broughhton, P. M. G. *et al., Journal of Clinical Pathology,* **22** (1969), 278.
8. Smith, J. *et al., Clinical Chemistry,* **28/9** (1982), 1867.
9. Tonks, D. B., *Canadian Journal of Medical Technology,* **30** (1968), 38.
10. Levin, G. E. *et al., An Evaluation of the Coulter Dacos TM* (1984), DHSS STB3A/84/26.

